# Diabetes-Related Topics in an Online Forum for Caregivers of Individuals Living With Alzheimer Disease and Related Dementias: Qualitative Inquiry

**DOI:** 10.2196/17851

**Published:** 2020-07-06

**Authors:** Yan Du, Kristi Paiva, Adrian Cebula, Seon Kim, Katrina Lopez, Chengdong Li, Carole White, Sahiti Myneni, Sudha Seshadri, Jing Wang

**Affiliations:** 1 Center on Smart and Connected Health Technologies School of Nursing The University of Texas Health Science Center at San Antonio San Antonio, TX United States; 2 School of Nursing The University of Texas Health Science Center at San Antonio San Antonio, TX United States; 3 School of Biomedical Informatics The University of Texas Health Science Center at Houston Houston, TX United States; 4 Glenn Biggs Institute for Alzheimer’s and Neurodegenerative Diseases The University of Texas Health Science Center at San Antonio San Antonio, TX United States

**Keywords:** diabetes, Alzheimer disease, dementia, caregivers

## Abstract

**Background:**

Diabetes and Alzheimer disease and related dementias (ADRD) are the seventh and sixth leading causes of death in the United States, respectively, and they coexist in many older adults. Caring for a loved one with both ADRD and diabetes is challenging and burdensome.

**Objective:**

This study aims to explore diabetes-related topics in the Alzheimer’s Association ALZConnected caregiver forum by family caregivers of persons living with ADRD.

**Methods:**

User posts on the Alzheimer’s Association ALZConnected caregiver forum were extracted. A total of 528 posts related to diabetes were included in the analysis. Of the users who generated the 528 posts, approximately 96.1% (275/286) were relatives of the care recipient with ADRD (eg, child, grandchild, spouse, sibling, or unspecified relative). Two researchers analyzed the data independently using thematic analysis. Any divergence was discussed among the research team, and an agreement was reached with a senior researcher’s input as deemed necessary.

**Results:**

Thematic analysis revealed 7 key themes. The results showed that comorbidities of ADRD were common topics of discussions among family caregivers. Diabetes management in ADRD challenged family caregivers. Family caregivers might neglect their own health care because of the caring burden, and they reported poor health outcomes and reduced quality of life. The online forum provided a platform for family caregivers to seek support in their attempts to learn more about how to manage the ADRD of their care recipients and seek support for managing their own lives as caregivers.

**Conclusions:**

The ALZConnected forum provided a platform for caregivers to seek informational and emotional support for caring for persons living with ADRD and diabetes. The overwhelming burdens with these two health conditions were apparent for both caregivers and care recipients based on discussions from the online forum. Studies are urgently needed to provide practical guidelines and interventions for diabetes management in individuals with diabetes and ADRD. Future studies to explore delivering diabetes management interventions through online communities in caregivers and their care recipients with ADRD and diabetes are warranted.

## Introduction

### Background

Diabetes and Alzheimer disease and related dementias (ADRD) are the seventh and sixth leading causes of death in the United States, respectively, and they coexist in many older adults [1,2]. Both diseases are major public health concerns in today’s aging world, and the combination of the two has further burdened individuals with the two diseases, their caregivers, and the society at large. As of 2019, 5.8 million Americans are estimated to live with Alzheimer dementia, and the population of people living with the disease is expected to triple by 2060 [3]. The total annual cost of managing and treating ADRD alone is estimated to be upward of US $215 billion and is expected to increase to US $500 billion by 2040 [3]. Likewise, diabetes affects roughly 30 million people in the United States as of 2015 and is expected to grow to affect approximately 55 million people between 2015 and 2030. Costs associated with the care and management of diabetes rose from US $245 billion in 2012 to US $327 billion in 2017 [2]. Together, the cost to care for persons with ADRD and diabetes comorbidity is higher than the cost to care for either disease alone. For example, the mean cost of diabetes care in persons with ADRD is approximately 3 times higher than the cost of diabetes care in those without ADRD [4].

A significant number of individuals living with ADRD have comorbid conditions, and diabetes is one of the most common comorbidities [5]. Increasing studies have documented that there is an association between ADRD and diabetes, and researchers have proposed that Alzheimer disease is *type 3 diabetes* because of the shared pathological mechanisms [6,7]. Moreover, the onset of diabetes may affect cognitive performance. For instance, higher levels of glycosylated hemoglobin and higher average blood glucose levels are correlated with poorer cognitive performance [8,9]. According to a longitudinal study, those who had type 2 diabetes at baseline demonstrated accelerated cognitive decline at 12-year follow-up [10]. Possible underlying mechanisms for this cognitive decline have been linked to insulin resistance, insulin-degrading enzyme, and so forth. Meanwhile, some studies suggest that there is a bidirectionality between the two diseases [11,12]. Previous studies have indicated that Alzheimer disease may affect the diabetic phenotype through behavioral changes, such as binge eating, or pathological changes, such as amyloid-beta deposition and tau protein phosphorylation [12].

### Caring for Individuals With ADRD and Diabetes

Although the mechanisms connecting these two diseases are still largely unknown, the implications are important as ADRD can affect the self-management of diabetes and vice versa. Both ADRD and diabetes require a unique and sometimes extensive amount of care. The management of diabetes alone involves constant blood glucose monitoring, the discipline to adhere to dietary restrictions and physical activity guidelines, and proper antidiabetic medication use [13]. Poor glycemic control can result in neuronal damage, increased ADRD incidence, and exaggerated cognitive function decline [12]. For those living with ADRD, the complexity of these regimens is often exacerbated by disease progression. Increased forgetfulness and confusion associated with the mid to late stages of dementia can result in improper meal choices or medication mismanagement [14]. This inability to participate in diabetes management as clinically instructed can result in harmful effects on the renal, cardiovascular, and peripheral nervous systems. As a result, people with ADRD may require assistance with the management of instrumental activities of daily living, such as diabetes management, from a caregiver.

Caring for persons with ADRD is often assumed by family members and close friends, with 83% of help to older adults in the United States being provided by these unpaid caregivers (referred to as family caregivers in this study) [1,15]. Recent estimates show that about 16 million family members and friends provided over 18 billion hours of unpaid care to people with ADRD at an average of 21.9 hours of care per week per caregiver [1]. This time spent caring for a loved one often comes at the expense of a caregiver’s own needs, with some sacrificing vacations, hobbies, and quality time with other family members [16]. In addition, the burden of caregiving is shown to be higher in caregivers of people with ADRD as compared with caregivers of older adults without ADRD, and the impact of this can be both physical and emotional [1,17]. For example, 40% of these family ADRD caregivers suffer from depression, and 1 in 3 family caregivers reported worsening health since assuming caregiving responsibilities [1]. Moreover, it has been shown that ADRD caregiving is associated with elevated biomarkers correlating to cardiovascular disease risk and that hormones associated with stress can have negative effects on glucose metabolism [18,19]. However, a recent study that reviewed 89 papers concluded that despite personalized, continuity, and family-centered care being urgently needed when caring for people living with ADRD and diabetes, current health care systems cannot meet the caring needs [20]. Individuals with ADRD and their caregivers constantly struggle with managing diabetes and ADRD.

To mitigate the burden associated with caring for a loved one with ADRD, some caregivers turn to social media to discuss patient care and their personal struggles as they adjust to their everchanging responsibilities as caregivers. For those living with diabetes, social media allows for additional communication and discussion outside of the limited time allotted with their physicians while encouraging participation and engagement [21]. Whether being used for the discussion of personal health or to discuss the care of a loved one, social media connects caregivers not only to like-minded individuals but also to health information and tools that may have otherwise been unavailable to them [22]. ALZConnected [23], a free online forum run by the Alzheimer’s Association in the United States, offers a range of forums for people with ADRD and their caregivers or friends and family members. For some, visiting this website means not having to explain what living with Alzheimer means, and for others, it means no longer feeling alone [23].

### Social Media Data

In this digital era, social media has provided platforms for users to seek health information, receive health interventions, and anonymously share thoughts and experiences that they may not feel confident or comfortable expressing in a real-world setting [24-27]. The increasing amount of user-generated information has become a valuable resource to guide health interventions and advance our knowledge of a variety of health conditions [28]. The data can be analyzed and built upon, allowing researchers to better understand user characteristics, social and information needs, and communication ecosystems as well as to serve the demographics being studied [29-32]. Such applications include, but are not limited to, the identification of individuals at risk for depression [33], the detection of drug-drug interactions or adverse side effects [34], the examination of health behaviors in various types of cancer survivors [35,36], and the investigation of the impact of social media on decision making and recovery in prostate cancer patients [37,38]. Whether the information gathered comes from well-known social media sites such as Facebook and Twitter or from more specific online support groups such as ALZConnected, the resulting analysis may provide much-needed insight to improve health outcomes in diverse groups [39-42]. However, despite the potential of media data to complement data from regulatory, clinical, administrative, and claims data sources, the utility and effectiveness of such data remain understudied in a variety of health conditions [43].

### Study Objective

This study aimed to explore diabetes-related topics in the ALZConnected caregiver forums by family caregivers of persons living with ADRD.

## Methods

### Setting/Study Population

In this qualitative study, we collected and analyzed posts from users of the Alzheimer’s Association ALZConnected caregiver’s forum in the United States [41]. The forum is designed to provide a community for ADRD caregivers and people with dementia alike. Caregivers, both professional and family, make up the majority of users in the forum and are often the spouse or child of the care recipient. Users must be registered in the ALZConnected community before they can post new threads or post in another user’s thread. Users must also be registered to view a member user’s profile. However, all posts can be viewed publicly without registering an account. The study was approved by the institutional review board of the University of Texas Health Science Center at San Antonio.

### Data Collection

**Figure 1:**
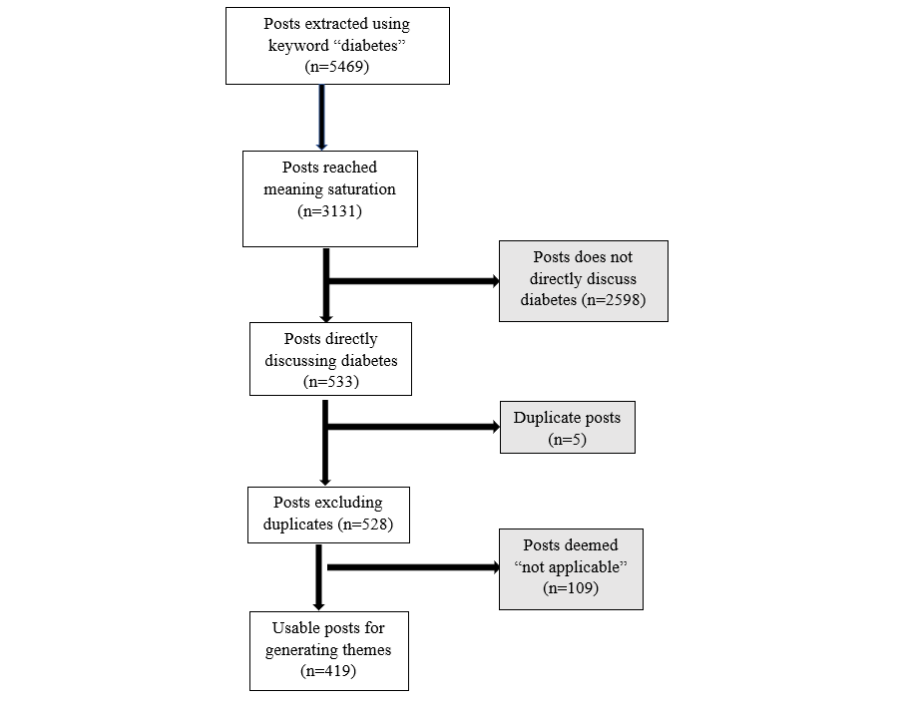
Flowchart of selection scheme of posts included in the study.

The earliest post in the forum was published on December 1, 2011. Posts were extracted from the forum backward in time via web scraping in January 2019. A total of 5469 user posts, posted from January 22, 2012, to January 3, 2019, were extracted using the keyword “diabetes.” All extracted posts were imported into an Excel (Microsoft) file for analysis, with password protection. Due to the nature of the data and the study focus of diabetes in individuals living with ADRD in an ADRD forum, further selection of posts and preliminary data analysis were conducted simultaneously. Two trained research assistants independently reviewed posts from the beginning of the list. Codes, notes, and comments were made during this process. Saturation was reached, meaning no new ideas were presented [44], after reviewing 3131 posts. Of the 3131 posts reviewed, 2598 were excluded for not directly discussing diabetes, leaving 533 posts. An additional 5 duplicate posts were removed, leaving 528 posts for analysis. Upon further analysis and the application of codes, an additional 109 were deemed *not applicable* because of the use of diabetes for comparative purposes or without reference to a care recipient or caregiver. Posts coded as *not applicable* were excluded, leaving 419 posts included in generating themes. Figure 1 illustrates the selection scheme of the posts.

The study also obtained publicly available data on the relationship between the post generator and individuals living with ADRD, aggregately. When registering for an account in the forum, users are requested to identify their relationship with a person living with dementia (eg, the user is a person living with dementia, a type of relative, or a professional caregiver).

### Data Analysis

“Thematic analysis is a method of identifying, analyzing, and reporting patterns (themes, topics, ideas) within a data” [45,46]. It has been widely used to identify themes or topics for web-based data (eg, blogs, web-based discussion boards, and tweets) [47-50] and data studying individuals living with ADRD [47,48,51]. Three trained research assistants analyzed the data using thematic analysis with the following steps [45,49,52]. First, data reduction and open coding were conducted during the preliminary analysis, and 2 coders developed a list of possible codes and definitions independently. The coding team and a senior qualitative scientist met to discuss and finalize the initial codes based on the feedback of all team members. Second, code validation was conducted by applying the initial codes to each of the 528 posts by 2 independent coders. Coders were instructed to code at the level of the post and were allowed to apply multiple codes to each post. Researchers coding overlapping sections of the dataset then came together in meetings to reconcile code differences in their respective analyses. They could discuss their reasoning for code applications. If in agreement, the 2 coders would combine code applications that served as the final analysis of the respective post being discussed. If the 2 coders were unable to reconcile code differences, the third coder provided insight allowing for a finalized analysis of the post. The initial codes from the first step were refined during the second step, and 26 distinct codes were generated. During this process, 109 were deemed *not applicable*, leaving 419 posts for the next step. Third, following coding reconciliation, the coding team displayed all various codes in a table and categorized them according to similarities and differences. Consideration was given to the coding processes, categorization, and noting patterns, and they came to a consensus of the key themes identified from the forum.

The coding team used descriptive statistics to obtain basic counts regarding the use of each code as well as to compare the number of instances in which 2 codes appeared together within the same thread. In addition, publicly accessible data of a user’s relationship with the individual living with ADRD were assessed aggregately using descriptive statistics.

## Results

### Caregiver-Patient Relationship

A total of 528 posts were generated by 286 unique users posting within 399 threads. Approximately 70% (200/286) of these users were the child of an individual with ADRD for whom they were caring for or concerned, whereas only 17.1% (49/286) of the users were spouses who were caring for a loved one with ADRD. Overall, the vast majority of these users (275/286, 96.1%) were related to the care recipient with ADRD in some way, that is, a child, grandchild, spouse, sibling, or unspecified relative. In contrast, very few users were professional caregivers (6/286, 2.1%) or someone living with ADRD (3/286, 1.0%).

### Themes

Thematic analysis revealed 7 key themes: (1) disease linkages and comorbidities, (2) ADRD or diabetes symptoms without diagnosis, (3) diabetes management, (4) ADRD and diabetes complications and progress, (5) management strategies for ADRD and other comorbidities, (6) caregiver social support, and (7) caregiver health and self-care. Of these, the first 5 are predominantly focused on the individual living with ADRD and the last 2 focus on the caregiver. Table 1 shows the themes, codes, and code frequency.

**Table 1:** Themes, codes, and code frequency.

Themes and codes		Code frequency, n
**Disease linkages and comorbidities**		
	ADRD^a^ and diabetes comorbidity	267
	ADRD and other comorbidities	123
	ADRD and diabetes link	107
**ADRD or diabetes symptoms without diagnosis**		
	Unconfirmed diagnosis of ADRD	46
	Diabetes differential diagnosis	35
**Diabetes management**		
	Diabetes medication management	124
	General diabetes management	109
	Diet and nutrition in diabetes management	107
	Physical activity and exercise	17
**ADRD and diabetes complications and progress**		
	Changes in ability and capacity	135
	Resistance	73
	Diabetes complications	55
	Forgetfulness	49
**Management strategies for ADRD and other comorbidities**		
	Obstacles to patient care coordination	200
	Pharmacotherapy	196
	Fiblets	31
	Diet (general)	28
	Alternative medicine	5
**Caregiver social support**		
	Informational support	233
	Caregiver burden	185
	Emotional support	156
**Caregiver health and self-care**		
	Caregiver self-care struggles	57
	Caregivers with diabetes	40
	Caregiver health outcomes	38
	Caregiver positive self-care	37

^a^ADRD: Alzheimer disease and related dementias.

### Disease Linkages and Comorbidities

This theme encompasses discussions on 2 or more diseases suffered by their care recipient. Within posts in this sample, it was common for caregiver to provide context for their care recipient’s situation by describing the health conditions of the care recipient. Posts falling under this theme either discussed ADRD and diabetes, ADRD and another comorbidity, or the proposed link between ADRD and diabetes. Forum participants would often describe the kind of ADRD experienced by the care recipient. Given that this sample was focused on diabetes, many individuals with ADRD also had diabetes. If other comorbidities were discussed (eg, heart disease and stroke), it was extremely likely that individuals with ADRD also had diabetes in addition to other comorbidities:

My dad has congestive heart failure, has had 2 heart attacks, multiple TIAs, a stroke, stage 4 kidney disease, COPD, high blood pressure, diabetes, and after we moved them in with us four years ago was diagnosed with mild vascular dementia.

To a lesser degree but still important to the discussion of diseases related to ADRD and diabetes was the link between the 2 diseases. Frequently, this was discussed in terms of disease pathology and the common link of insulin resistance to both diseases. Users would speculate as to how strong the connection was between the 2 diseases and if one might lead to the other. Some users would also share links to articles to support their claims:

I myself find most compelling the connection between the top three listed and Alzheimer’s type dementia: These include: insulin resistance, diabetes, and obesity; These three are very related anyway.

Here is a high level overview of the diabetes/Alzheimer’s connection... Bottom line watch that sugar closely! This is a decades long thing. https://tinyurl.com/y842evt8.

### ADRD or Diabetes Symptoms Without Diagnosis

Forum use was not limited to individuals with dementia and their caregivers. This theme encompasses discussions on a care recipient who had some symptoms of ADRD or diabetes but had not been formally diagnosed. Individuals questioning symptoms that a loved one was experiencing utilized the forum to ask users if the symptoms or behaviors could be a sign of ADRD. In some cases, a caregiver of an individual with a diagnosis of ADRD also questioned whether pre-existing conditions (eg, diabetes) might be the root cause of cognitive changes being displayed by a care recipient, rather than a diagnosis of ADRD. It was common for these forum participants to seek information about the process of diagnosis and how to work with their loved one to receive medical care:

Can anyone suggest a way to find a specialist who sincerely look at my mom's situation and make sure she is being correctly diagnosed with dementia. No one has really done the proper testing to rule out possible mimics and I have not gotten an exact diagnoses of the type. I have been to 2 neurologist and I feel they just do the verbal test and say she has dementia. What about b12 deficiencies or the fact that she is on blood pressure medicine.

Respondents to these forum participants, at times, provided a differential diagnosis based on the symptoms a user describes. Given the similar pathology of ADRD and diabetes (as well as other diseases), it is possible that the symptoms a user described as possible dementia were diabetes:

With someone your dad's age, it is important to complete the testing because there are over 50 other illnesses that look like Alz but are not and most importantly of all, many of these illnesses can be reversed. Certainly mismanagement of diabetes can cause cognitive changes, even in young people.

### Diabetes Management

Within this theme, caregivers discussed the challenges they face (eg, balancing diabetes management and quality of life) and shared some practical strategies they use when helping an individual living with ADRD to manage his or her diabetes. Overall, there were frequent discussions of diet and medications used to manage diabetes. Although some of these posts focused on individuals who had good management of diabetes, it was common for caregivers to question whether adherence to diabetes management practices was necessary for those with advanced ADRD. Caregivers expressed feelings that the overall quality of life was more important than strict adherence; however, they still maintained that diabetes management was important. Thus, caregivers commonly discussed how best to maintain a good quality of life for the care recipient, first and foremost, with a more relaxed approach to managing diabetes:

Mom, a type 2 diabetic, loves sweets...At some point in the last few months I decided that if having access to colas would give mom a 5 star day instead of a 3 star day and she should have the cola...I want to balance between having a good quality of life and a (somewhat) healthy life.

It was also frequent for forum respondents to discuss the lack of diabetes management and high or variable blood sugar readings, which an individual with ADRD might experience:

His blood sugar was over 1000! The hospital got it under control and recommended that he have his blood sugars checked four times a day and given oral medicine and injected insulin to control it. The problem is that he doesn't want to get up in the morning or the afternoon and he is refusing to eat frequently which he must do to take the insulin.

In contrast, very few caregivers discussed physical activity or exercise as a part of diabetes management for their care recipient. When they did, the discussion often centered around a concern for a lack of physical activity on the part of the care recipient:

I would like to ask for some advice on managing my father's repetitive behaviors. For example, he will sit idle for HOURS, sometimes ENTIRE days just staring off into space in the dark...My concern is the sitting idle for hours. He has diabetes, is in 3rd stage kidney failure, has poor circulation, and muscles that are getting VERY weak from being sedentary.

Given the complexity of managing diabetes in an ADRD care recipient, caregivers frequently discussed management strategies for their care recipients as well. For example, some caregivers helped care recipients to manage their diabetes through a diet with inventive approaches such as halving juice with water, providing Meals on Wheels subscriptions, or slowly exchanging poor food choices with more healthy alternatives. Caregivers often noted that these changes had to be made slowly, as abrupt changes often resulted in resistance from their care recipients:

My mom would report cereal with OJ for breakfast...When I got over there, it turned out that was true, so I started a Meals on Wheels subscription for her and dad. One meal was diabetes health labelled with her name and one was heart healthy labeled with his name...With orange juice, what we did was save one of the empty containers of orange juice and then pour half into the empty container and dilute the OJ by half. Mom never noticed the difference.

However, some caregivers felt that a more direct approach better served their care recipients and, instead, opted to obtain the medical power of attorney or hire a home health professional to manage the care recipient’s diabetes themselves. Whether their strategies were direct or indirect, caregivers frequently discussed practical ways to encourage or enforce adherence to diabetes management regimens through medication or diet:

Auditory memory is poor, she's repetitious and we are concerned about her continuing to live alone because she is an insulin dependent diabetic... I fill her pillbox and monitor it as much as possible, but I know that there are days when she doesn't take some of her pills...I have POA for healthcare and other POA is in the works.

### ADRD and Diabetes Complications and Progress

This theme encompasses discussions on how an ADRD care recipient’s resistance or forgetfulness complicates care management. Many caregivers came to the forum to discuss disease progress and disease complications in their care recipients. Often forum users discussed the changes in abilities or capacities that an individual living with ADRD experienced and how this complicated overall care. Many of these posts expressed a worsening of disease state, which ultimately led to a change in the caregiver’s caregiving experience:

My mother has been having real issues with her memory for well over a year now. It has gotten progressively worse...We have been lengthening her visits over the past year to help her adjust to the idea of living with us.

When discussing complications and progress, some caregivers spoke about how resistance to diagnosis and forgetfulness affected the ability of the caregiver to provide appropriate care to care recipients for diabetes and ADRD, as it complicated how they provided care or how a care recipient responded to care:

I think my biggest concern about my mom continuing alone is the impact that her short term and auditory memory issues have on her medication management, more than anything else. My mom doesn't chart her blood sugars, never has. That's just who she is. If I ask her if she ate or took insulin, she doesn't remember.

He has declined rapidly by his own choices not to follow the doctors orders from the very beginning. He won't monitor his blood sugars properly and rarely administers his insulin because he doesn't like the pricking of his finger or the pain of the shot. He eats whatever he wants and it consists entirely of carbs, high fats and sugar.

Although less frequent, some of the users described complications of diabetes as one of the many concerns that their care recipient was experiencing:

The consequences of untreated diabetes are not fun; things like blindness and amputations of toes, then feet, then below the knee...I am sure your LO [loved one] does not want those, and yet with dementia they cannot make rational choices based on future risk.

### Management Strategies for ADRD and Other Comorbidities

Although this sample focused on diabetes, caregivers came to this forum, most importantly, to discuss ADRD. Within this theme, caregivers discussed how they helped an individual living with ADRD and with the management of comorbidities other than diabetes. As a result of the complexities caregivers experienced in caring for an individual with ADRD, caregivers frequently had questions or provided advice about how to navigate the housing, medical, legal, and day-to-day needs of their loved one:

After much discussion with my grandmother we have decided that my grandfather should be put in a nursing home. I unfortunately don't know a lick of anything related to senior care. I have been doing nonstop research and am so confused...I know some nursing homes take medicare but I am extremely weary.

It is time for you to step in and take control of the situation. You must protect her. I am hoping that you are already your mother's financial and medical POA. If not, you need to contact your family attorney. If your family doesn't have an attorney, then look for an eldercare attorney in your area.

A common management technique was using medication to help manage ADRD or other comorbidities that the individual with ADRD might be experiencing:

My mother was on Namenda 10mg once a day for two and a half years. I made the decision to stop the meds last June after many discussions (over time) with her PCP...My mom is currently taking Risperidone to manage anger and agitation and has been for two years. She also takes oral meds to manage Type II Diabetes and high blood pressure.

However, caregivers also had unconventional techniques to help them manage the complexities of multiple conditions for an individual living with ADRD. For example, some caregivers would use a *fiblet* or a therapeutic lie to help a patient to adhere to a medical regimen, a diet change, or new living arrangements:

In the beginning we did tell mom and she was always shocked: “I have Alzheimers?!?!” Now I just say her blood sugars levels are too high (she's diabetic) and once we can get control on them, she'll feel better. It's a lie, I know, but I don't see the point in continually telling her.

Occasionally, caregivers discussed the use of special diets, such as the Mediterranean diet, to prevent the worsening of ADRD. This was distinct from the use of diet to manage diabetes and blood glucose levels:

...The “grain brain” as a cause of dementia may be somewhat off because many grains also contain polyphenols that help protect against Alzheimer's disease such as ferulic acid in rice bran or flaxseed in some breads. That is why a Mediterranean diet relatively high in carbohydrates is partially protective against Alzheimer's disease.

### Caregiver Social Support

A prominent theme within the forum was social support. Individuals frequently used the forum to seek support related to providing care or to provide support to other individuals in need of information or encouragement. Many users discussed the burden of taking care of a patient with ADRD and would use the forum to discuss frustrations or stress associated with caregiving as well as to seek support:

I struggle with the what if's even though everything says AD. Being her daughter, I want to throw her in the vehicle, drive her to the doctor and have them check her from head to toe everyday. But at what expense? Her dignity? Her comfort? I do everything I know to do and well...I just wish I guess...I feel like I'm rambling out of exhaustion.

Some users also provided emotional support to help encourage a user who was struggling:

I'm sorry to hear about what you are going through with your family. For myself, as someone on the outside looking in, the solutions seem easy, but it isn't.

In addition, users posed questions and gave advice that was strictly informational in nature. Some users provided guidance and suggestions derived from their personal experiences, and others provided links, articles, and phone numbers for resources that they believed would be valuable to the individual with the question:

To answer your main question - how do we convince our LO to do certain things that will help them? Well, that usually doesn't work because even if they are agreeable to the idea, their brains don't retain new information or learn new habits very well if at all. As far as my mom, for a long time we went back and forth with the idea of her living with me...mom had POA in order but had “capacity” (a psychiatrist evaluated her and found her able to make her own decisions) so I couldn't force anything.

I would like to invite you to contact the Helpline at the Alzheimer's Assn. which can be reached at: (800) 272-3900…Consultants are highly educated Social Workers who specialize in dementia and they can be wonderful support, provide education materials, have contact numbers for helpful community entities and who can assist with problem solving and more.

### Caregiver Health and Self-Care

In contrast to social support, this theme was focused on discussions on caregivers’ own physical and mental health and self-care. However, some caregivers did discuss having their own diagnosis of diabetes or another health concern:

Anxiety has now joined it, and Dad is now in the nh [nursing home]. But caring for Dad here at home for 12 years triggered it - along with hypertension and prediabetes. These are all stress related health issues.

In discussions of their own health outcomes, some caregivers discussed their inability to participate in self-care activities. This struggle to maintain a healthy lifestyle, whether physically or mentally, was often attributed to a lack of free time because of the sheer amount of time spent caregiving:

There are times, I am so exhausted that I just want to “drop dead”. I am too tired of being tired...too tired to rest. But I keep moving forward.

I have no life at all, haven't seen friends in years, can't establish any new friends, can't set up any type of routine for my own enjoyment or benefit (school, hobbies, gym, martial arts) or work on my own life that I walked away from to take care of her and I am always exhausted these days.

In contrast, others were concerned about developing chronic diseases, such as dementia and diabetes, and participated in and promoted positive self-care behaviors to reduce their risk of developing diseases:

I say this as a caregiver who also has trouble taking care of myself sometimes, but life is forcing me to learn. In fact, right now, I need to get off the computer and get outside for some exercise, which is my daily medicine against encroaching diabetes, heart disease, and mental illness from all the stress.

My next health challenge is to NOT get dx with diabetes, so over the past few months I've stopped stress-eating chocolate foods, and have finally started exercising daily after the Mom-details, which took many months to sort out, are sorted.

## Discussion

### Principal Findings

This is, to the best of our knowledge, the first study to examine diabetes-related topics in individuals with ADRD from the perspectives of family caregivers participating in an online support community. Our findings advance knowledge regarding the role of social media in health management. The findings are consistent with previous findings that health forums provide support to forum users (eg, informational and emotional support) [53,54] and offer platforms for users in communication of diseases in general, disease symptoms and treatments, and user opinions [55]. However, unlike other health forum users, most of whom are patients themselves [27,56], the ALZConnected forum was designed for caregivers. The study findings demonstrated how family caregivers used this online forum to obtain resources for informational and emotional support and to seek ways to relieve caregiving burden stress. As this study focused on diabetes-related topics, discussions of the analyzed posts were always related to (1) health conditions of care recipients, symptoms, and management of diabetes and ADRD; (2) the daily burden and struggles of diabetes management in individuals with both ADRD and diabetes, which might not be captured by traditional data collection; and (3) how constant caregiver burden and daily struggles affect their self-care and result in poorer physical and mental health in family caregivers.

Forum participants extensively discussed information related to the care recipients’ health conditions with comorbidities, links between ADRD and comorbidities, and uncertainty of the root causes of some symptoms and behaviors. This may reflect the complex interactions of ADRD and its comorbidities [57] and the underlying causes of family caregivers’ struggles. Older adults without dementia have 2 comorbidities on average, whereas those living with dementia have an average of 4 comorbidities, and almost 9 out of 10 individuals living with dementia have at least one comorbidity [58]. A scoping review found that little is known about the care of comorbidities for people living with dementia and comorbidities, especially from the perspectives of those with dementia and their family caregivers [59]. Our study further echoes and reinforces the importance of understanding the management of ADRD comorbidities, especially diabetes, from the experience of family caregivers. This line of discussion in the forum could serve as an outlet for forum users to relieve their frustrations and anxiety about the uncertainty of the disease progression in their care recipients living with multiple chronic comorbidities and how they can better care for their loved ones. Moreover, the information obtained from the forums by caregivers might also prove useful in supporting communication with their health providers, both for themselves and their care recipients.

Given the focus on diabetes in this study, we found that family caregivers commonly discussed challenges associated with diabetes management in their loved ones living with ADRD, regardless of type 1 or type 2 diabetes. For example, many challenges faced by caregivers centered on diet, exercise, and medication management in their care recipients. This is unsurprising, as diabetes is largely a self-managed disease that necessitates high-level cognitive capabilities for patients to properly adhere to medication and lifestyle regimens. However, memory loss, impaired problem-solving, and other ADRD symptoms make diabetes management extremely difficult for people living with ADRD and their family caregivers [14,60]. Previous recommendations and studies have suggested that for people with cognitive impairment, diabetes care should be individualized and patient-centered [60]. The glycemic target (eg, hemoglobin A_1c_, fasting glucose, and postprandial glucose) should also be relaxed [14]. However, the incorporation of these recommendations into daily care regimens has rarely been studied in individuals living with ADRD and diabetes. In fact, studies examining diabetes self-management often exclude individuals with cognitive impairment. In addition, despite the well-recognized importance of including family caregivers in the development of self-management plans for ADRD, few studies have evaluated structured interventions to provide education and support for family caregivers of ADRD [60], and even less studies have focused on diabetes management. Studies involving people living with ADRD and their family caregivers are urgently needed to address diabetes *self-management* in home settings for this population living with both ADRD and diabetes.

For challenges of diabetes management, the findings also highlight the difficulties caregivers face when trying to balance quality of life and diabetes control in their ADRD care recipients. Much of this is associated with the responsibility of providing proper health care to their loved ones that caregivers feel while still making sure their loved one’s overall quality of life is high. Regardless of a caregiver’s best efforts, uncontrolled blood glucose levels and subsequent hospital admission were often discussed by family caregivers. Balancing glycemic control and quality of life in ADRD care recipients warrants further exploration. Despite how challenges were frequently discussed, in the forum, some personal practical strategies for diabetes management were also shared. For example, some caregivers helped care recipients manage their diabetes through gradual diet changes or with the use of inventive approaches, such as halving juice with water. Whether the shared strategies were direct or indirect, caregivers frequently discussed practical ways by which they encouraged and enforced adherence to diabetes management regimens, whether through medication or diet. The various strategies shared may serve as examples for forum users looking to tailor the approaches to their own loved ones and situations. In addition, these suggestions might provide helpful information and examples for developing diabetes management guidelines for people living with ADRD and their family caregivers. This further reinforces the importance of involving caregivers and their loved ones in studies of developing diabetes management guides.

Our study revealed that forum users, who take care of individuals with ADRD and diabetes, frequently reported increased distress and neglect of self-care, both of which may contribute to a decrease in health status and quality of life for both the care recipient and family caregivers [61]. Previous studies reported that the time spent on daily activities and supervision by caregivers was higher when caring for individuals with ADRD and diabetes compared with caring for those with ADRD without diabetes [62]. Consequently, the level of the caregiver’s perceived burden increases [63-66]. ALZConnected has provided a platform for family caregivers to seek information and emotional support in their attempts to learn more about how to manage ADRD of their care recipients and seek support for how to manage their own health and well-being as a caregiver. The study findings of challenges and struggles that caregivers constantly face have implications not just on their care recipients but also on their own health and well-being. We call for more practical diabetes management guidelines for individuals living with ADRD and their caregivers to ease the burden of caregivers and improve the health and quality of life for both.

### Limitations

This study has several limitations. First, the study used publicly available forum data, and as a result, the data collection process was out of the control of the study team. However, this could also be considered a strength as there was no interference with participants’ opinions, meaning the posts reviewed truly reflect the caregivers’ struggles and concerns. Second, the posts extracted for this study only represent a portion of posts made in the forum and may not be applicable to all discussions regarding diabetes. However, our analysis did reach saturation, which might minimize this limitation. Third, we attempted to explore discussion topics related to diabetes in this forum and used thematic analysis to analyze the data [67]. Thus, there is no theoretical framework to support this study. Fourth, this study only used data from one social media platform, and the discussions in the forum are largely user-driven. In the future, using multiple social media data sources may further extend our knowledge of the study topic. Finally, a majority of the posts included in this study were from adult children of ADRD care recipients, which might reflect their proportion of family care or that younger persons are more likely to use social media/online forums. As a result, the generalizability of the results to all family caregivers might be limited.

### Conclusions

In summary, the overwhelming burdens of diabetes management in individuals living with ADRD and diabetes were apparent for both caregivers and care recipients based on discussions from the ALZConnected forum. Research involving both care recipients and their caregivers in developing diabetes management guidelines and interventions for family caregivers of individuals with diabetes and ADRD would be important. In addition, the ALZConnected forum provided a platform for caregivers to seek informational and emotional support for caring for persons living with ADRD and diabetes. Studies are urgently needed to provide practical guidelines or tools available in online support communities as new ways to support daily diabetes management for individuals living with ADRD coexisting with diabetes and their caregivers. Future studies to explore delivering diabetes management interventions through online communities in caregivers and their care recipients with ADRD and diabetes are warranted.

## Abbreviations

ADRD: Alzheimer disease and related dementias

## References

[1] (2019). 2019 Alzheimer’s Disease Facts and Figures. Alzheimer's Association: Alzheimer's Disease & Dementia Help.

[2] (2020). National Diabetes Statistics Report 2020. Centers for Disease Control and Prevention.

[3] (2018). Alzheimer's Disease. Centers for Disease Control and Prevention.

[4] Salber PR, Selecky CE, Soenksen D, Wilson T (2018). Impact of dementia on costs of modifiable comorbid conditions. Am J Manag Care.

[5] Bunn F, Burn AM, Goodman C, Robinson L, Rait G, Norton S, Bennett H, Poole M, Schoelman J, Brayne C (2016). Comorbidity and dementia: a mixed-method study on improving health care for people with dementia (CoDem). Health Serv Delivery Res.

[6] Kandimalla R, Thirumala V, Reddy PH (2017). Is Alzheimer's disease a type 3 diabetes? A critical appraisal. Biochim Biophys Acta Mol Basis Dis.

[7] Mittal K, Mani RJ, Katare DP (2016). Type 3 diabetes: cross talk between differentially regulated proteins of type 2 diabetes mellitus and Alzheimer's disease. Sci Rep.

[8] Ravona-Springer R, Heymann A, Schmeidler J, Moshier E, Godbold J, Sano M, Leroith D, Johnson S, Preiss R, Koifman K, Hoffman H, Silverman JM, Beeri MS (2014). Trajectories in glycemic control over time are associated with cognitive performance in elderly subjects with type 2 diabetes. PLoS One.

[9] Cukierman-Yaffe T, Gerstein HC, Williamson JD, Lazar RM, Lovato L, Miller ME, Coker LH, Murray A, Sullivan MD, Marcovina SM, Launer LJ, Action to Control Cardiovascular Risk in Diabetes-Memory in Diabetes (ACCORD-MIND) Investigators (2009). Relationship between baseline glycemic control and cognitive function in individuals with type 2 diabetes and other cardiovascular risk factors: the action to control cardiovascular risk in diabetes-memory in diabetes (ACCORD-MIND) trial. Diabetes Care.

[10] Spauwen PJ, Köhler S, Verhey FR, Stehouwer CD, van Boxtel MP (2013). Effects of type 2 diabetes on 12-year cognitive change: results from the Maastricht aging study. Diabetes Care.

[11] Roberts RO, Knopman DS, Przybelski SA, Mielke MM, Kantarci K, Preboske GM, Senjem ML, Pankratz VS, Geda YE, Boeve BF, Ivnik RJ, Rocca WA, Petersen RC, Jack CR (2014). Association of type 2 diabetes with brain atrophy and cognitive impairment. Neurology.

[12] Shinohara M, Sato N (2017). Bidirectional interactions between diabetes and Alzheimer's disease. Neurochem Int.

[13] American Diabetes Association (2003). Standards of medical care for patients with diabetes mellitus. Diabetes Care.

[14] Puttanna A, Padinjakara NK (2017). Management of diabetes and dementia. Br J Diabetes.

[15] Prince M, Albanese E, Guerchet M, Prina M (2014). Dementia and Risk Reduction: An Analysis of Protective and Modifiable Factors. Alzheimer's Disease International.

[16] Schulz R, Martire LM (2004). Family caregiving of persons with dementia: prevalence, health effects, and support strategies. Am J Geriatr Psychiatry.

[17] Schulz R, Sherwood PR (2008). Physical and mental health effects of family caregiving. Am J Nurs.

[18] von Känel R, Mills PJ, Mausbach BT, Dimsdale JE, Patterson TL, Ziegler MG, Ancoli-Israel S, Allison M, Chattillion EA, Grant I (2012). Effect of Alzheimer caregiving on circulating levels of C-reactive protein and other biomarkers relevant to cardiovascular disease risk: a longitudinal study. Gerontology.

[19] Surwit RS, Schneider MS (1993). Role of stress in the etiology and treatment of diabetes mellitus. Psychosom Med.

[20] Bunn F, Goodman C, Jones PR, Russell B, Trivedi D, Sinclair A, Bayer A, Rait G, Rycroft-Malone J, Burton C (2017). Managing diabetes in people with dementia: a realist review. Health Technol Assess.

[21] Gabarron E, Bradway M, Fernandez-Luque L, Chomutare T, Hansen AH, Wynn R, Årsand E (2018). Social media for health promotion in diabetes: study protocol for a participatory public health intervention design. BMC Health Serv Res.

[22] Anderson JG, Hundt E, Dean M, Keim-Malpass J, Lopez RP (2017). 'The church of online support': examining the use of blogs among family caregivers of persons with dementia. J Fam Nurs.

[23] (2014). ALZConnected.

[24] Robinson J, Cox G, Bailey E, Hetrick S, Rodrigues M, Fisher S, Herrman H (2016). Social media and suicide prevention: a systematic review. Early Interv Psychiatry.

[25] Welch V, Petkovic J, Pardo Pardo J, Rader T, Tugwell P (2016). Interactive social media interventions to promote health equity: an overview of reviews. Health Promot Chronic Dis Prev Can.

[26] Zhao Y, Zhang J (2017). Consumer health information seeking in social media: a literature review. Health Info Libr J.

[27] Sinha A, Porter T, Wilson A (2018). The use of online health forums by patients with chronic cough: qualitative study. J Med Internet Res.

[28] Martí P, Serrano-Estrada L, Nolasco-Cirugeda A (2019). Social media data: challenges, opportunities and limitations in urban studies. Comput Environ Urban.

[29] Liu D, Yamashita T, Burston B (2018). Identifying consumers who search for long-term care on the web: latent class analysis. JMIR Aging.

[30] Mikal JP, Grande SW, Beckstrand MJ (2019). Codifying online social support for breast cancer patients: retrospective qualitative assessment. J Med Internet Res.

[31] Shang L, Zuo M, Ma D, Yu Q (2019). The antecedents and consequences of health care professional-patient online interactions: systematic review. J Med Internet Res.

[32] Signorelli GR, Lehocki F, Fernández MM, O'Neill G, O'Connor D, Brennan L, Monteiro-Guerra F, Rivero-Rodriguez A, Hors-Fraile S, Munoz-Penas J, Dalmau MB, Mota J, Oliveira RB, Mrinakova B, Putekova S, Muro N, Zambrana F, Garcia-Gomez JM (2019). A research roadmap: connected health as an enabler of cancer patient support. J Med Internet Res.

[33] Karmen C, Hsiung RC, Wetter T (2015). Screening internet forum participants for depression symptoms by assembling and enhancing multiple NLP methods. Comput Methods Programs Biomed.

[34] Vilar S, Friedman C, Hripcsak G (2018). Detection of drug-drug interactions through data mining studies using clinical sources, scientific literature and social media. Brief Bioinform.

[35] Olsen A, Keogh J, Sargeant S (2019). Investigating how bowel cancer survivors discuss exercise and physical activity within web-based discussion forums: qualitative analysis. J Med Internet Res.

[36] Lognos B, Carbonnel F, Launay IB, Bringay S, Guerdoux-Ninot E, Mollevi C, Senesse P, Ninot G (2019). Complementary and alternative medicine in patients with breast cancer: exploratory study of social network forum data. JMIR Cancer.

[37] de Silva D, Ranasinghe W, Bandaragoda T, Adikari A, Mills N, Iddamalgoda L, Alahakoon D, Lawrentschuk N, Persad R, Osipov E, Gray R, Bolton D (2018). Machine learning to support social media empowered patients in cancer care and cancer treatment decisions. PLoS One.

[38] Johansson P, Westas M, Andersson G, Alehagen U, Broström A, Jaarsma T, Mourad G, Lundgren J (2019). An internet-based cognitive behavioral therapy program adapted to patients with cardiovascular disease and depression: randomized controlled trial. JMIR Ment Health.

[39] Eliya Y, Pellegrini D, Gevaert A, Code J, van Spall HG (2019). Social media in heart failure: a mixed methods systematic review. Curr Cardiol Rev.

[40] Fisher S, Jehassi A, Ziv M (2019). Hidradenitis suppurativa on Facebook: thematic and content analyses of patient support group. Arch Dermatol Res.

[41] (2019). Discussion Board: Caregivers Forum. ALZ Connected.

[42] Rolls K, Hansen M, Jackson D, Elliott D (2016). How health care professionals use social media to create virtual communities: an integrative review. J Med Internet Res.

[43] Tricco AC, Zarin W, Lillie E, Jeblee S, Warren R, Khan PA, Robson R, Pham B, Hirst G, Straus SE (2018). Utility of social media and crowd-intelligence data for pharmacovigilance: a scoping review. BMC Med Inform Decis Mak.

[44] Hennink MM, Kaiser BN, Marconi VC (2017). Code saturation versus meaning saturation: how many interviews are enough?. Qual Health Res.

[45] Castleberry A, Nolen A (2018). Thematic analysis of qualitative research data: is it as easy as it sounds?. Curr Pharm Teach Learn.

[46] Braun V, Clarke V (2006). Using thematic analysis in psychology. Qual Res Psychol.

[47] Cheng TY, Liu L, Woo BK (2018). Analyzing twitter as a platform for Alzheimer-related dementia awareness: thematic analyses of tweets. JMIR Aging.

[48] Clark IN, Tamplin JD, Baker FA (2018). Community-dwelling people living with dementia and their family caregivers experience enhanced relationships and feelings of well-being following therapeutic group singing: a qualitative thematic analysis. Front Psychol.

[49] Morris MT, Daluiski A, Dy CJ (2016). A thematic analysis of online discussion boards for brachial plexus injury. J Hand Surg Am.

[50] Sullivan CF (2003). Gendered cybersupport: a thematic analysis of two online cancer support groups. J Health Psychol.

[51] Greenwood N, Smith R, Akhtar F, Richardson A (2017). A qualitative study of carers' experiences of dementia cafés: a place to feel supported and be yourself. BMC Geriatr.

[52] Alhojailan M (2012). Thematic analysis: a critical review of its process and evaluation. West East J Soc Sci.

[53] Ito T, Meguro K, Akanuma K, Ishii H, Mori E (2007). A randomized controlled trial of the group reminiscence approach in patients with vascular dementia. Dement Geriatr Cogn Disord.

[54] Evans M, Donelle L, Hume-Loveland L (2012). Social support and online postpartum depression discussion groups: a content analysis. Patient Educ Couns.

[55] Pérez-Pérez M, Pérez-Rodríguez G, Fdez-Riverola F, Lourenço A (2019). Using Twitter to understand the human bowel disease community: exploratory analysis of key topics. J Med Internet Res.

[56] Litchman ML, Edelman LS, Donaldson GW (2018). Effect of diabetes online community engagement on health indicators: cross-sectional study. JMIR Diabetes.

[57] Subramaniam H (2019). Co-morbidities in dementia: time to focus more on assessing and managing co-morbidities. Age Ageing.

[58] Poblador-Plou B, Calderón-Larrañaga A, Marta-Moreno J, Hancco-Saavedra J, Sicras-Mainar A, Soljak M, Prados-Torres A (2014). Comorbidity of dementia: a cross-sectional study of primary care older patients. BMC Psychiatry.

[59] Bunn F, Burn A, Goodman C, Rait G, Norton S, Robinson L, Schoeman J, Brayne C (2014). Comorbidity and dementia: a scoping review of the literature. BMC Med.

[60] Bunn F, Goodman C, Reece Jones P, Russell B, Trivedi D, Sinclair A, Bayer A, Rait G, Rycroft-Malone J, Burton C (2017). What works for whom in the management of diabetes in people living with dementia: a realist review. BMC Med.

[61] Varela G, Varona L, Anderson K, Sansoni J (2011). Alzheimer's care at home: a focus on caregivers strain. Prof Inferm.

[62] Lebrec J, Ascher-Svanum H, Chen Y, Reed C, Kahle-Wrobleski K, Hake AM, Raskin J, Naderali E, Schuster D, Heine RJ, Kendall DM (2016). Effect of diabetes on caregiver burden in an observational study of individuals with Alzheimer's disease. BMC Geriatr.

[63] Cheng S (2017). Dementia caregiver burden: a research update and critical analysis. Curr Psychiatry Rep.

[64] Chiao C, Wu H, Hsiao C (2015). Caregiver burden for informal caregivers of patients with dementia: a systematic review. Int Nurs Rev.

[65] Haro JM, Kahle-Wrobleski K, Bruno G, Belger M, Dell'Agnello G, Dodel R, Jones RW, Reed CC, Vellas B, Wimo A, Argimon JM (2014). Analysis of burden in caregivers of people with Alzheimer's disease using self-report and supervision hours. J Nutr Health Aging.

[66] Mioshi E, Foxe D, Leslie F, Savage S, Hsieh S, Miller L, Hodges JR, Piguet O (2013). The impact of dementia severity on caregiver burden in frontotemporal dementia and Alzheimer disease. Alzheimer Dis Assoc Disord.

[67] Vaismoradi M, Turunen H, Bondas T (2013). Content analysis and thematic analysis: implications for conducting a qualitative descriptive study. Nurs Health Sci.

